# DOPS Adjuvant Confers Enhanced Protection against Malaria for VLP-TRAP Based Vaccines

**DOI:** 10.3390/diseases6040107

**Published:** 2018-11-21

**Authors:** Gustavo Cabral-Miranda, Ahmed M. Salman, Mona O. Mohsen, Federico L. Storni, Elisa S. Roesti, Murray A. Skinner, Matthew D. Heath, Matthias F. Kramer, Shahid M. Khan, Chris J. Janse, Adrian V. S. Hill, Martin F. Bachmann

**Affiliations:** 1 The Jenner Institute, Nuffield Department of Medicine, Centre for Cellular and Molecular Physiology (CCMP), Roosevelt Drive, Oxford OX3 7BN, UK; ahmed.salman@ndm.ox.ac.uk (A.M.S.); mona.mohsen@dbmr.unibe.ch (M.O.M.); adrian.hill@ndm.ox.ac.uk (A.V.S.H.); 2 Department of Immunology, RIA, Inselspital, University of Bern, Sahlihaus 1/2, 3010 Bern, Switzerland; federico.storni@dbmr.unibe.ch (F.L.S.); elisa.roesti@dbmr.unibe.ch (E.S.R.); 3 Bencard Adjuvant Systems, Dominion Way, Worthing BN14 8SA, UK; Murray.Skinner@allergytherapeutics.com (M.A.S.); Matthew.Heath@allergytherapeutics.com (M.D.H.); KramerM@bencard.com (M.F.K.); 4 Department of Parasitology, Leiden University Medical Center, Albinusdreef 2, 2333 ZA Leiden, The Netherlands; s.m.khan@lumc.nl (S.M.K.); c.j.janse@lumc.nl (C.J.J.)

**Keywords:** vaccine, adjuvants, *Plasmodium falciparum*, malaria, virus like particle (VLP), dioleoyl phosphatidylserine (DOPS)

## Abstract

Vaccination remains the most effective and essential prophylactic tool against infectious diseases. Enormous efforts have been made to develop effective vaccines against malaria but successes remain so far limited. Novel adjuvants may offer a significant advantage in the development of malaria vaccines, in particular if combined with inherently immunogenic platforms, such as virus-like particles (VLP). Dioleoyl phosphatidylserine (DOPS), which is expressed on the outer surface of apoptotic cells, represents a novel adjuvant candidate that may confer significant advantage over existing adjuvants, such as alum. In the current study we assessed the potential of DOPS to serve as an adjuvant in the development of a vaccine against malaria either alone or combined with VLP using *Plasmodium falciparum* thrombospondin-related adhesive protein (TRAP) as a target antigen. TRAP was chemically coupled to VLPs derived from the cucumber mosaic virus fused to a universal T cell epitope of tetanus toxin (CuMVtt). Mice were immunized with TRAP alone or formulated in alum or DOPS and compared to TRAP coupled to CuMVtt formulated in PBS or DOPS. Induced immune responses, in particular T cell responses, were assessed as the major protective effector cell population induced by TRAP. The protective capacity of the various formulations was assessed using a transgenic *Plasmodium berghei* expressing PfTRAP. All vaccine formulations using adjuvants and/or VLP increased humoral and T cell immunogenicity for PfTRAP compared to the antigen alone. Display on VLPs, in particular if formulated with DOPS, induced the strongest and most protective immune response. Thus, the combination of VLP with DOPS may harness properties of both immunogenic components and optimally enhance induction of protective immune responses.

## 1. Introduction

Malaria remains a massive challenge for global health [1,2,3]. According to the World Health Organization (WHO), the occurrence of 214 million cases and 438,000 deaths caused by malaria occurred worldwide a few years ago [4]. Five plasmodium species exist that cause malaria in humans, but only two of them dominate disease: *Plasmodium falciparum* and *Plasmodium vivax* [5,6]. Despite these impressive numbers, up to the present time there is no licensed malaria vaccine for worldwide use in humans. The flag-ship vaccine against *P. falciparum* RTS,S/AS01, based on the circumsporozoite (CSP) antigen, is licensed in a limited way only, mostly due to sometimes disappointing efficacy in clinical trials [7]. The RTS,S vaccine is well-known to induce strong antibody and Th cell responses; however induction of CD8^+^ T cells is usually weak. More recently, an optimized version of RTS,S, called R21 has entered clinical development with some exciting new properties in preclinical [8] and clinical trials (unpublished data). R21 comprises only the hybrid CS-HBsAg protein in the VLP with no additional HBsAg monomers. Nevertheless, overall efficacy may be improved with additional antigens and adjuvants able to induce strong T cells immunity may also be beneficial for this purpose.

Many antigens have been tested in vaccine development, and a promising, relatively recent, malaria vaccine candidate is the thrombospondin-related adhesive protein (TRAP), a transmembrane protein with extracellular adhesive domains essential for sporozoite motility and liver cell invasion [9]. TRAP is thought to be mainly a target for T cell-based vaccines and may therefore an important addition to those vaccines that already provide protective antibody response. In addition to CD8^+^ T cells, some studies also indicate an importance of TRAP-specific antibodies as protective effector mechanism against malaria [10,11,12,13].

Even the best potentially protective antigen usually requires the use of a potent adjuvant or adjuvant system to help focus and develop the immune response to act faster, stronger, and offer long-term immunity, without compromising safety. Adjuvants can modulate the type of immune cells triggered and enhance the overall immune response [14]. Since the beginning of the 20th century, when the first adjuvants (aluminum salts) were tested in humans, the concept of adjuvants has become an important component of vaccine development and is now a key target for improving immunogenicity and efficacy of modern vaccines [15]. Aluminum-based preparations (usually summarized under the term “alum”) remain the most commonly used adjuvants in both human and veterinary vaccines [16,17], but evidence of the better effectiveness of novel adjuvants for existing and emerging diseases is continuing to grow.

Alum-based adjuvants have a huge safety record and positive benefit risk profile outweigh potential concerns in the context of prophylactic vaccination programs. Alum is cost-effective, easy to manufacture, and licensure of alum formulated products is comparably straightforward. Moreover, alum has been used effectively in adjuvant systems, combining alum with immunostimulators [18]. However, some more difficult-to-target pathogens, such as malaria, may benefit from a more potent combination of adjuvants that in particular, would induce more potent Th1 rather than Th2 responses as well as CD8^+^ T cell responses. In addition, alum tends to induce less protective IgG subclasses, such as IgG1 in mice or IgG4 in humans. Hence, if protection is not solely based on pathogen neutralization, but Fc-receptor interactions and complement are also important, alternative adjuvants may be more potent than alum [17,19,20,21,22,23].

Phosphatidylserine derivatives are part of a family of related compounds that may differ in their fatty acid content. They are distributed within the inner leaflet of plasma membranes. During apoptosis PS derivatives are translocated to the outer membrane where it functions as a surface signaling molecule, recognized by phagocytic cells such as macrophages [24]. PS derivatives, including Di-oleoyl-phosphatidyl-serine (DOPS), have been shown to modulate ovalbumin-specific antibody production in different ways. In these experiments, DOPS significantly increased the production of IgG subclasses, with no significant effect on IgE production [25,26]. 

Antigens displayed on VLPs are repetitive and benefit from intrinsic adjuvant properties of VLPs, resulting in strong B cell responses [27,28]. In addition, VLPs, such as the here employed CuMVtt VLP package RNA, which drives Th1 and IgG2a responses in a TLR7/8-dependent manner. Here we demonstrate that combination with DOPS, but not alum, further enhances these properties yielding optimal protection upon vaccination with a TRAP-CuMVtt-based vaccine. Hence, we present a formulation that is built on established pharmaceutical principles combining drug delivery concepts (VLP) in combination with a second generation adjuvant (immunomodulator) functioned to provide a means by which immune activation could be better achieved for a vaccine to confer enhanced protection against malaria.

## 2. Material and Methods

### 2.1. PfTRAP Protein Production

The PfTRAP gene (accession number: PF3D7_1335900) was synthesized by Geneart™ with a His.tag sequence of six histidine amino acids (HHHHHH) at the C-terminal and subsequently cloned into an expression vector pHLsec at the AgeI and KpnI restriction sites. The new construct AS-807 = PfTRAP-His.tag in pHLsec was transformed into *Escherichia coli* DH5a bacteria and the growth of bacterial cultures was purified using QIAGEN Plasmid Mega Kit. The pHLsec-PfTRAP purified plasmid was transfected into HEK-293T cells for protein production. The supernatant from HEK-293T cells culture was purified from dialyzed conditioned medium against phosphate-buffered saline (PBS) buffer by immobilized nickel affinity chromatography, followed by size-exclusion chromatography in 20 mM Tris-HCl, pH 8.0, and 300 mM NaCl.

### 2.2. Vaccines Preparation

The VLP used to prepare the vaccine by coupling with PfTRAP protein was derived from the cucumber mosaic virus fused to a universal T cell epitope of the tetanus toxin (CuMVtt) that was expressed in *E. coli* BL21 (DE3) Star (Thermo Fisher Scientific) and purified as previously described [10]. The PfTRAP protein was covalently conjugated to VLP using the strategy already published [10]. The conjugated VLP-PfTRAP vaccine was analyzed by SDS-PAGE and Western blot to detect bands corresponding to the various components of the coupling reaction. The PfTRAP protein was also used to prepare vaccine based on aluminum hydroxide adjuvant (alum) (InvivoGen, San Diego, USA) and phosphatidyl serine (PS) derivatives—1,2-dioleoyl-sn-glycero-3-phospho-L-serine (DOPS) (Avanti Polar Lipids, Inc., Alabaster, Alabama, USA).

The vaccine formulation using alum was prepared using 10 µg of Al^3+^ per dose per mouse according to the manufacturer’s specifications (InvivoGen, San Diego, CA, USA). The alum was mixed to PfTRAP protein and vortexed gently for few seconds and incubated at room temperature for one hour and subsequently vaccinate the mice.

In order to generate PS-DOPS formulated candidate vaccines, PS-DOPS was dissolved in PBS and mixed to PfTRAP protein or VLP-PfTRAP vaccine and gently vortexed for few seconds. Vaccine batches were freshly produced for each vaccination. 

### 2.3. Immunization Protocol

In order to check the vaccine efficacy, 6 female BALB/c inbred mice per group and 8 female CD1 outbred mice per group, 6 weeks old, purchased from Harlan, United Kingdom, were vaccinated intramuscularly (i.m.) with 20 µg (50 μL) per mouse of PfTRAP protein diluted in PBS, mixed with alum or DOPS adjuvants, chemically coupled to VLP with or without DOPS. The vaccinations were done 3 times: prime (Day 0), first boost (Day 21), and second boost (Day 42). The samples (blood) were collected before each vaccination on Days 0, 21, 42, also on Days 50 and 59 to check the humoral and cellular immunogenicity. On Day 63, three weeks after the second boost, the mice were challenged with a transgenic *P. berghei* replacement expressing PfTRAP protein (mutant # 2632 cl1). The determination of prepatent period (after challenge of immunized mice with transgenic sporozoites) was checked by counting the level of parasitemia in the blood smears daily beginning on fourth day after the challenge until the mice reach 1% parasitemia. The mice that did not show any erythrocytes infected until 14th day after challenge were considered sterile protected.

### 2.4. Kinetics of Antibody Production

For assessment of antibody production, total IgG and its subclass, enzyme-linked immunosorbent assays (ELISAs) were performed. For that, 96-well microtiter ELISA plates (Thermo Scientific, Nottingham, UK) were coated with 100 µL per well in a concentration of 1 µg/mL of purified PfTRAP and diluted in carbonate buffer (CBB) 50 mM at pH = 9.6 and incubated overnight at 4 °C. In the next day the plates were filled with 200 μL of 2% BSA-PBS to avoid nonspecific binding and incubated at room temperature for two hours. The next step, the sera from mice were diluted in 0.2% BSA-PBS buffer, initially starting with 1 in 100 and followed by eleven 1/3 serial dilution in the ELISA plates. For the total IgG measurement, 100 μL per well of goat anti-mouse IgG diluted to 1:2000 (Secondary Antibody, HRP conjugate (ThermoFisher, Paisley, UK) were added and incubated for 1 h at room temperature. For assessment of IgG subclass, goat anti-mouse IgG subclass (goat anti-mouse IgG1, IgG2a, IgG2b HRP coupled, Life Technologies) were used in a dilution of 1:2000, and incubated for 1h at room temperature. Between all the previous steps the ELISA plates were washed by filling the wells four times with 200 µL PBS—0.05% Tween and 200 µL PBS. To develop the reaction, 100 μL/well of TMB substrate (Sigma-Aldrich) was applied and incubated at RT for 10 min and under aluminum foil for light protection. After that, the reaction was stopped by adding 100 μL/well of 0.5 M H_2_SO_4_ (sulfuric acid) and the plates read using microplate reader at 450 nm. The titers are expressed as dilutions leading to half-maximal OD (OD50).

### 2.5. Assessment of TCD4 Cells Polarization

The evaluation of CD4^+^ T cell polarization was made by detection of TNFα, IFNγ (Th1 cytokines) and IL-4 (Th2 cytokine) using sera collected on Day 50 (1 week after the second boost) by multiplex assays. The goal was to determine whether differences in serum cytokine profile exist between unvaccinated and vaccinated mice with PfTRAP coupled to VLP with or without DOPS adjuvants, as well as to compare the adjuvants DOPS vs. alum in term of Th1 and Th2 cytokines stimulations. The methodology applied was that described by MHSTCMAG-70K kit guidelines of MILLIPLEX MAP Mouse High Sensitivity T Cell Panel. Briefly, all reagents from the kit were brought to room temperature and vortexed before adding to the plate provided with the kit. After that, the antibody-immobilized beads were prepared in the mixing bottle, as well as the quality controls, serum matrix and standards. The sera from vaccinated and nonvaccinated mice were defrosted completely and spin to avoid particulates. The sera samples were diluted 1:2 using assay buffer and used 25 µL per well, two wells per sample. The immunoassay procedure consisted adding 50 µL of standard 1–7 and 50 µL of serum matrix to background (standard 0). Followed by adding 25 µL assay buffer to sample wells, plus 25 µL serum to all sample wells. Twenty-five microliters of vortexed beads were added to all wells (standard and mice sera), plate sealed, and incubated overnight at 4 °C on rocker. The following day the immunoassay procedure was established using Magpix PC, by creating a standard with input cytokine concentrations as per assay to calculate the average of these and what is the expected. Moreover, sample details are added with their dilution factors. After this, the plate was agitated on plate shaker (450 rpm) for 10 mins at room temperature, and then ready to perform the analysis. A quality control step was then performed, to check the file was created correctly for each cytokine and review standard curves that must within their expected range for each cytokine.

### 2.6. Evaluation of CD8^+^ T Cells by Intracellular Cytokine Staining (ICS)

The CD8^+^ T cell response was assessed by ICS, detecting specific IFNγ and TNFα production in stimulated peripheral blood mononuclear cells (PBMC). For that, PBMC on Day 50 (1 week after the second boost) were collected from vaccinated and naïve mice. The PBMC cells were stimulated with immunodominant Pb9 peptide for 12 h with a concentration of 1 µg/ml [29], as well as brefeldin A (BFA) (BD GolgiPlug™). To check the cell viability, aqua live/dead cell staining (Thermo Fisher Scientific) was added. The CD3^+^ T cells were stained using the antibodies anti-mouse CD3 Alexa Fluor^®^ 700 (Clone: 17A2), anti-mouse CD8 conjugated with PE (Clone: 53-6.7), anti-mouse tumor necrosis factor alpha (TNFα) APC (Clone: MP6-XT22), and anti-mouse interferon-gamma (IFNγ) (Clone: XMG1.2). For the flow cytometry valuation was used a BD FACSCanto™ Flow Cytometer (BD Biosciences, Oxford, UK). For data analysis the software FlowJo (Flowjo, Ashland, OR, USA) or GraphPad Prism (Graphpad Software, Inc., San Diego, CA, USA) were applied. 

### 2.7. Animals

Female inbred BALB/c (H-2^d^) and outbred CD1 (ICR) mice were used for the assessment of immunogenicity and protection after challenge. Tuck-ordinary (TO) outbred mice were used for parasite production and transmission. Mice were purchased from Harlan (UK). Transgenic parasites were developed in Leiden University Medical Centre (LUMC) using 6-week old Swiss mice (Charles River).

### 2.8. Ethics Statement

All animals and procedures were used in accordance with the terms of the UK Home Office Animals Act Project License. Procedures were approved by the University of Oxford Animal Care and Ethical Review Committee (PPL P9804B4F1). Animal experiments performed at LUMC were approved by the Animal Experiments Committee of the Leiden University Medical Center (DEC 12042). The Dutch Experiments on Animals Act was established under European Guidelines (EU Directive no. 86/609/EEC regarding the Protection of Animals used for Experimental and Other Scientific Purposes).

### 2.9. Parasite Production

Wild type and transgenic parasites used to challenge mice were produced at the insectary of the Jenner Institute. Female *Anopheles stephensi* mosquitoes were fed on infected BALB/c mice. Briefly, exflagellation was first confirmed and mosquitoes were exposed to anaesthetized infected mice for 10 min. Mosquitoes were then maintained for 21 days in a humidified incubator at a temperature of 19–21 **°**C on a 12 h day–night cycle and fed with a fructose/PABA solution.

### 2.10. Parasites

The wild type (WT) reference line cl15cy1 of *P. berghei* ANKA [30] and the reporter *Pb*ANKA parasite line *Pb*GFP-Luc_con_ (676m1cl1). *Pb*GFP-Luc_con_ parasite expresses a fusion protein of GFP (mutant3) and firefly luciferase (LUC-IAV) under the constitutive *eef1a* promoter and is SM-free [31]. The reporter-cassette is integrated into the neutral *230p* locus (PBANKA_030600). For details of *Pb*GFP-Luc_con_, see RMgmDB entry #29 (http://www.pberghei.eu/index.php?rmgm=29).

### 2.11. Generation of DNA Constructs and Genotyping of the Transgenic Parasites

To generate the transgenic parasites where the *P. berghei trap* gene (PBANKA_134980) coding sequence (CDS) has been replaced by the CDS of *P. falciparum trap* (PF3D7_1335900), we used a 2-step GIMO transfection protocol [31,32]. In the first step we deleted the *P. berghei trap* CDS and replaced it with the positive-negative selectable marker, to create a *P. berghei trap* deletion GIMO line (PbANKA-TRAP GIMO). In order to this we generated pL2111 construct that is based on the standard GIMO DNA construct pL0034 [31]. This construct contains the positive-negative (h*dhfr*:y*fcu)* selection marker (SM) cassette, and was used to insert both the *Pbtrap* 5′ and 3′ gene targeting regions (TR), encompassing the full length promoter and transcription terminators sequences respectively. The construct was linearized using ApaI and NotI restriction enzymes outside of the 5′ and 3′ TRs before transfection. The linear pL2111 DNA construct was introduced into *Pb*GFP-Luc_con_ parasites using standard methods transfection [30]. Transfected parasites were selected in mice by applying positive selection by providing pyrimethamine in the drinking water [31]. Transfected parasites were cloned by limiting dilution [33], resulting in the PbANKA-TRAP GIMO line (2564 cl3) (Figure S1). Correct deletion of the *P. berghei csp* CDS was confirmed by diagnostic PCR-analysis on gDNA and Southern analysis of pulsed field gel (PFG)-separated chromosomes as described [30]. Primers used for PCR genotyping are listed in Table S1. 

In the second step we replaced the positive-negative SM in the PbANKA-TRAP GIMO genome with the CDS of *P. falciparum trap* by GIMO transfection to create the *P. berghei* transgenic TRAP replacement lines. This was achieved by modifying the construct used in the first step (pL2111) to generate pL2127 DNA construct; specifically, the h*dfhr*:y*fcu* SM cassette was removed and replaced with *P. falciparum trap* CDS sequence (Figure S1). The *P. falciparum trap* CDS was amplified by PCR from *P. falciparum* wild-type genome and was sequenced to ensure there were no mutations in the *P. falciparum trap* CDS. The construct was linearized using ApaI and KasI restriction enzymes outside of the 5′ and 3′ TRs before transfection. This construct was used to transfect parasites of the PbANKA-TRAP GIMO line (2564 cl3) using standard methods of GIMO-transfection [34]. Transfected parasites were selected in mice by applying negative selection by providing 5-fluorocytosine (5-FC) in the drinking water of mice [35]. Negative selection results in selection of chimeric parasites where the h*dhfr*:y*fcu* SM in the *trap* locus of PbANKA-TRAP GIMO line is replaced by the CDS of *P. falciparum* TRAP. Selected transgenic parasite was cloned by the method of limiting dilution [34]. Correct integration of the constructs into the genome of chimeric parasites was analyzed by diagnostic PCR-analysis on gDNA and Southern analysis of pulsed field gel (PFG) separated chromosomes as previously described [30]. Primers sequences used for generation of DNA constructs are shown in Table S2, and primers used for PCR genotyping are listed in Table S1, while the expected PCR product sizes and the primer numbers are listed in the table below (Table S3) the PCR analysis (Figure S2). This method creates transgenic ‘gene replacement’ *P. berghei* parasites that do not contain *P. berghei trap* gene CDS but express *P. falciparum* TRAP (PbANKA-PfTRAP(r)_PbTRAP_; 2632 cl1) under the control of the *P. berghei trap* regulatory sequences. 

### 2.12. Phenotyping of Reporter and Transgenic Parasites. 

Growth of blood stages of the reporter and transgenic *P. berghei* parasites was determined during the cloning period as described [30,34]. Feeding of *A. stephensi* mosquitoes, determination of oocyst production, and sporozoite collection were performed as previously described [34]. Expression of *PfTRAP* antigen in sporozoites of the transgenic parasites was analyzed by immunofluorescence-staining assay (IFA), using serum from vaccinated mice with PfTRAP or PbTRAP as a control; diluted 50 times. Purified sporozoites were fixed with 4% paraformaldehyde in PBS for 20 min on ice, then washed three times with PBS and blocked with 20 µL 10% FCS + 1% BSA in PBS for 30 min at room temperature. The excess blocking medium was removed, followed by the addition of 20 to 25 μL of the diluted serum containing the primary antibody in 10% FCS + 1% BSA in PBS (blocking medium) for 1 to 2 h at room temperature or overnight at 4 °C. After incubation the primary antibody was removed and the slides washed three times with PBS, followed by the staining with the secondary antibody (Alexa Fluor^®^ 488 Goat Anti-Mouse IgG from life technologies, Cat# A-11001) diluted 800 times in 10% FCS + 1% BSA in PBS (blocking medium) for one hour at room temperature. After washing three times with PBS, nuclei were stained with 2% Hoechst-33342 (Cell Signaling Technology #4082S) in PBS for 10 min at room temperature, washed twice with PBS and left to air-dry, this followed by adding Fluorescence Mounting Medium (Dako, code S3023) before complete dry out. Cover slips were mounted onto the slides, and the slides were sealed with nail polish and left to dry overnight in dark. The parasites in both blue and green channels were analyzed using a DMI-300B Leica fluorescence microscope and images processed using ImageJ software (Figure S3).

### 2.13. Efficacy Studies: Determination of Liver Parasite Liver Load by Real Time Imaging and Determination Prepatent Period (after Challenge of Immunized Mice with Transgenic Sporozoites)

To determine the efficacy of the liver-stage vaccines, transgenic *P. berghei* infected *A. stephensi* mosquitoes were dissected 21 days post-feed and salivary gland sporozoites resuspended in RPMI-1640 media (Sigma Aldrich). One-thousand transgenic sporozoites were injected i.v. into the tail vain per mouse, into both vaccinated and naïve controls (Figure S4).

### 2.14. Statistical Analysis and Parasitemia Prediction

Parasitemia levels in mice infected with *P. bergei-PfTRAP* were checked daily until reaching infection of 1% in blood-stage. For the parasitemia prediction a linear regression model was used as previously described [10,19,36,37,38]. In summary, blood from infected mice were collected daily after the fourth day post challenge and blood smears were stained with Giemsa and 1000 blood cells counted for each mouse sample. The logarithm to base 10 for percentage of parasitemia was plotted against the time after challenge and Prism 5 for Mac OS X (GraphPad Software, La Jolla, CA, USA) was used for generating a linear regression model on the linear part of the blood-stage growth curve. For all other statistical analyses, GraphPad Prism software version 5.0 for Max OS was also used. For the comparison of two normally distributed groups it was used unpaired *t*-test, although for comparing of two non-parametric groups a Mann–Whitney rank test was applied. When more than two groups were existing, non-parametric data was compared using Kruskal–Wallis test with Dunn’s multiple comparison post-test, whereas normally distributed data were analyzed by one-way ANOVA with Bonferroni’s multiple comparison post-test. The value of *p* < 0.05 was considered statistically significant (* *p* < 0.01, ** *p* < 0.001, *** *p* < 0.0001).

## 3. Results

### 3.1. DOPS Promotes a Th1 Response in TRAP-Vaccinated BALB/c Mice

**Figure 1:**
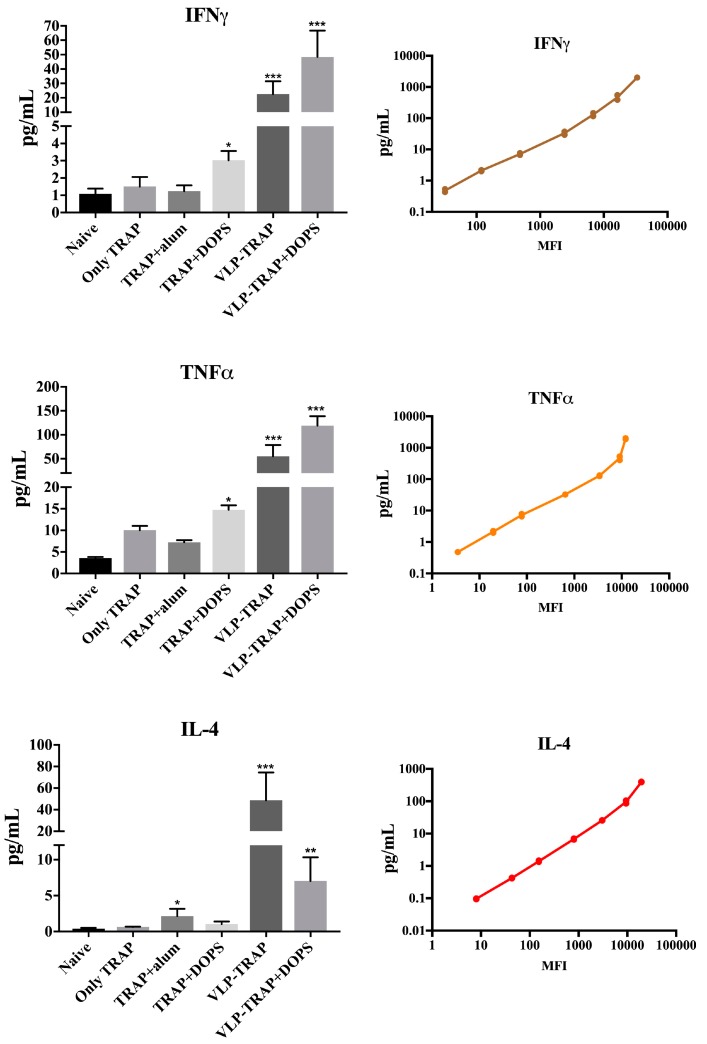
Assessment of CD4^+^ T cells polarization. Mice were immunized once every three weeks. One week after the third vaccination the mice were bled to obtain sera for cytokine analysis. To evaluate cytokine production, TNFα, IFNγ (Th1 and CTL), and IL-4 (Th2) were measured by multiplex assays MHSTCMAG-70K kit. Also, for experimental control, standard curves were made of each cytokine and compared with the expected concentration provided by the kit, as seen in the figure. The results were analyzed using GraphPad Prism software applied to assess the means of the groups by unpaired t-tests. Note: *** *p*-value < 0.0001; ** *p*-value < 0.001; * *p*-value < 0.01. MFI = Median Fluorescence Intensity.

The most used adjuvants in vaccine formulation for infectious diseases remain aluminum based. However, alum is inefficient at inducing Th1 immunity as responses are normally biased towards Th2 cytokines. However, for diseases such as malaria, vaccine formulations that induce Th1 responses are certainly preferred. In addition, induction of CD8^+^ T cells is also important, another characteristic that may not be typical for alum [17,19,20,21,22,23]. We therefore wanted to address, whether DOPS may be able to induce Th1 responses and aimed to directly compare the adjuvants activity of DOPS to alum. To this end, BALB/c mice were vaccinated i.m. with recombinant TRAP protein from *P. falciparum* formulated in alum, DOPS or PBS on days 0, 21, and 42. For assessment of Th1 and Th2 cell induction, serum samples from one week after the third immunization were collected and checked by multiplex assay. As seem in the Figure 1, those cytokines related to Th1 (IFNγ and TNFα) were increased in mice vaccinated with TRAP + DOPS compared to mice vaccinated with TRAP + alum or TRAP formulated in PBS. In contrast, IL-4 production was increased in mice vaccinated with TRAP + alum compared to mice vaccinated with TRAP + DOPS. Thus, as perhaps expected, alum induced a cytokine response dominated by IL-4 while DOPS induced a cytokine responses dominated by IFNγ and TNFα. 

TRAP was also displayed on CuMVtt-VLPs and tested with or without DOPS. Conjugation of antigens to VLPs containing RNA usually enhances Th1 responses and may represent an important step within the system to drive immune responses towards Th1. We therefore conjugated TRAP to CuMVtt and vaccinated BALB/c mice with CuMVtt-TRAP, with CuMVtt-TRAP + DOPS or only TRAP protein. As in the previous experiment, we assessed presence of Th1 and Th2 cytokines in the serum one week after the third boost. As seen in the Figure 1, mice vaccinated with CuMVtt-TRAP in PBS exhibited increased serum levels of IFNγ, TNFα, and IL-4. In contrast, CuMVtt-TRAP formulated with DOPS adjuvants exhibited highest serum levels of IFNγ and TNFα paralleled by a decrease in the IL-4 response. These findings support use of DOPS in combination with VLPs for the development of vaccines against malaria and other infectious diseases, as they require Th1 rather than Th2 responses. 

### 3.2. Antibody Production by Vaccinated BALB/c Mice

**Figure 2:**
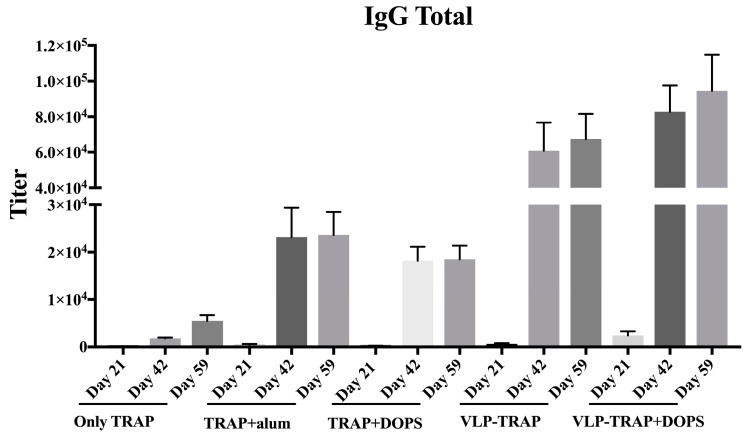
Kinetics of antibody production in BALB/c mice. The figure shows IgG total of all groups of mice bled three weeks after each vaccination, specifically on Days 21, 42, and 59. As seen in the figure, the mice that best responded were those vaccinated with PfTRAP coupled with VLP. Also, when the VLP-PfTRAP was mixed with DOPS adjuvants, it provided an improvement of the antibody response with highly statistical significant (*p* = 0.0001) when compared with the control group.

Protection induced by vaccination against TRAP is thought to be based on induction of T cell responses. However, some studies claim that antibodies against TRAP are also important and have to be considered when developing TRAP-based Vaccines. We therefore assessed the humoral immunogenicity of all groups mentioned above. As seen in Figure 2, after a single vaccination and also subsequently, the best responder group was vaccinated with CuMVtt-TRAP + DOPS. Thus, CuMVtt formulated in DOPS induced the strongest Th1 as well as antibody response. 

**Figure 3:**
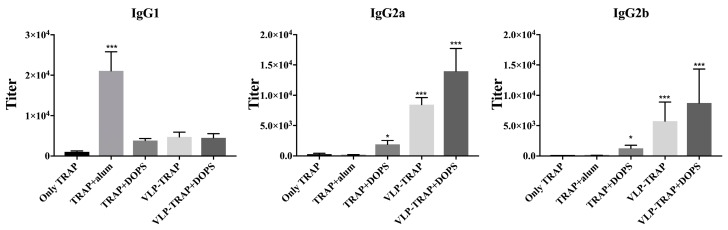
Assessment of IgG subtype in BALB/c mice. The antibody subclasses were tested on day 59 of the immunized mice. VLP-conjugate vaccines induced the more protective isotypes IgG2a and IgG2b compared to TRAP formulated in alum. In other hand, the group of mice vaccinated with TRAP + alum induced IgG1 subtype, as expected. Note: *** *p*-value < 0.0001; * *p*-value < 0.01.

The subclass of the induced antibodies is important as many studies show that IgG2a and IgG2b are the most protective subclasses in animal models. Also in humans, the IgG3 subclass (usually associated with IgG2b in mice) has been associated with protection against malaria [39,40,41,42,43,44,45]. As shown in Figure 3, the group vaccinated with TRAP formulated in alum exhibited IgG1 as the dominant IgG subclass. In contrast, the group vaccinated with TRAP formulated with DOPS alone or in combination with VLP predominantly produced IgG2a and IgG2b. TRAP formulated in DOPS, in particular when associated to CuMVtt, induced IgG responses dominated by the most protective subclasses, IgG2a and IgG2b.

### 3.3. TRAP-CuMVtt+DOPS Induces Best Protection Against Infection with P. Bergei Expressing PfTRAP

**Figure 4:**
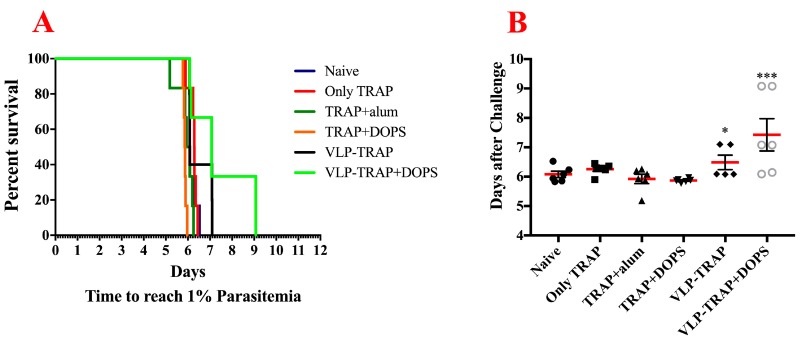
Survival curve and comparative analysis of parasitemia in BALB/c mice infected with *Plasmodium berghei* replacement expressing TRAP of P. falciparum (PfTRAP). (**A**) Kaplan–Meyer survival curve that shows the time when the mice reach 1% or more of the erythrocytes infected. (**B**) Number of days until mice have more than 1% of erythrocytes infected and the comparison of the groups. The experiment did not induce sterile protection, but immunization with PfTRAP-VLP plus DOPS conferred significant protection (*p* = 0.0005) when compared to the group control vaccinated with TRAP only. Also, infection in mice vaccinated with PfTRAP-VLP was significantly delayed when compared to the group vaccinated with only TRAP (*p* = 0.0354). *** *p*-value < 0.0001; * *p*-value < 0.01.

The most important and stringent way to assess the ability of vaccine formulations to induce protective immune responses is to assess actual protection against infection. However, *P. falciparum* does not infect mice. We therefore, constructed a transgenic *P. berghei* replacement mutant expressing TRAP of *P. falciparum* instead of its own protein. This transgenic parasite showed efficient capacity to infect and replicate in mice. To test vaccine efficacy, BALB/c mice were immunized with TRAP alone, TRAP formulated in alum or DOPS or TRAP-CuMVtt alone or formulated in DOPS. Two weeks after the third vaccination, immunized and naïve mice were infected with the transgenic *P. berghei* expressing PfTRAP. After infection, mice were assessed for parasite load daily until 1% of all erythrocytes were infected. At this point, mice had to be euthanized according to the terms of United Kingdom Home Office Animals Act Project License. As seen in the Figure 4 the most resistant group of mice was the one vaccinated with VLP-TRAP + DOPS. The delay of parasitemia seen in these mice correlates with the fact that they mounted the best immune response and shows that TRAP coupled to CuMVtt formulated in DOPS might be a promising vaccine candidate. 

### 3.4. Assessing the Efficacy of Vaccine Formulated with TRAP-VLP + DOPS in CD1 Mice

**Figure 5:**
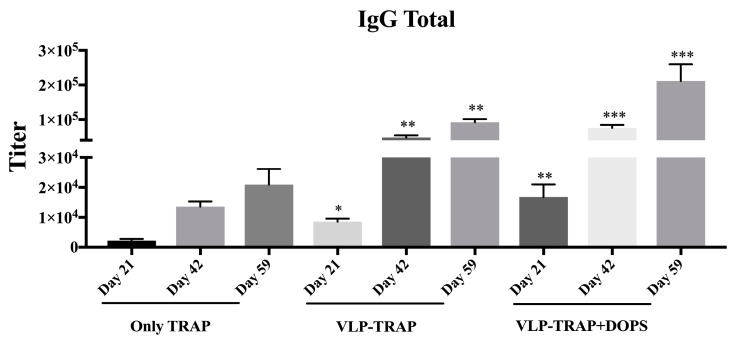
Kinetics of antibody production in CD1 mice. CD1 mice were immunized once every three weeks with TRAP, VLP-TRAP, or VLP-TRAP formulated with DOPS. Blood was taken on Days 21, 42, and 59 and serum antibody levels were determined. Total IgG levels against TRAP are shown in this figure; the group of mice vaccinated with PfTRAP coupled with VLP, as well as when the VLP-PfTRAP was mixed with DOPS adjuvants induced significantly antibody response when compared with the control group. Note: *** *p*-value < 0.0001; ** *p*-value < 0.001; * *p*-value < 0.01.

BALB/c is a well-tested mouse model for malaria vaccine development. However, it is important to test candidate vaccine formulations with different murine strains, in particular also outbred stains. For this reason, we confirmed the protective capacity of the most promising vaccine formulations (CuMVtt-TRAP + DOPS and CuMVtt-TRAP in PBS compared to TRAP alone) in CD1 outbred mice. As seen above in BALB/c mice, the humoral immune response mounted by CD1 mice vaccinated with VLP-TRAP + DOPS was higher than the group of mice vaccinated with TRAP-VLP without adjuvant, as well as the mice vaccinated with TRAP only (Figure 5).

**Figure 6:**
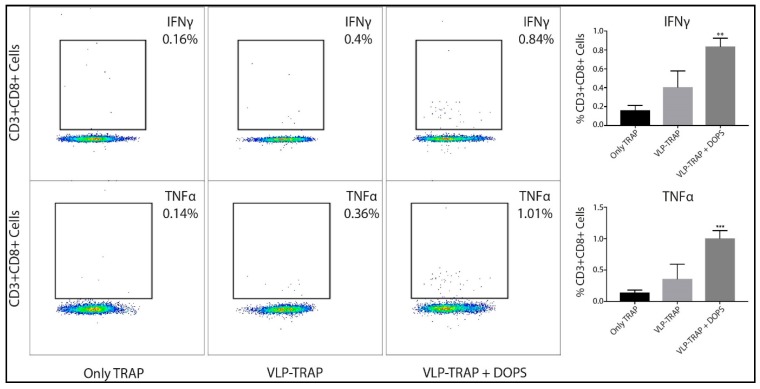
Assessment of TRAP-specific CD8^+^ T cell responses in CD1 mice. As described in Figure 1, mice were immunized three times every three weeks and bled on day 50 (eight days after the third vaccination) and the PBMC cells were stimulated with immunodominant Pb9 peptide to assess production of IFNγ and TNFα by CD8^+^ T cells. As seen in the figure above, the group of mice vaccinated with VLP-TRAP + DOPS was able to produce more IFNγ and TNFα than the groups of mice vaccinated with TRAP protein either VLP-TRAP. Note: *** *p*-value < 0.0001; ** *p*-value < 0.001.

In addition to humoral immunity, cellular immune responses were also assessed. The most advanced vaccine against malaria is the RTS,S formulated in AS01, which induces strong and protective antibodies as well as CD4^+^ T cell responses but fails to induce significant CD8^+^ T cell responses. We therefore assessed CD8^+^ T cell responses in mice vaccinated with the different TRAP formulations (Figure 6). TRAP coupled to CuMVtt VLPs formulated in DOPS induced the strongest CD8^+^ T cell responses, indicating that this vaccine formulation may be a promising addition to RTS,S/AS01, as it further promotes cellular immunity.

**Figure 7:**
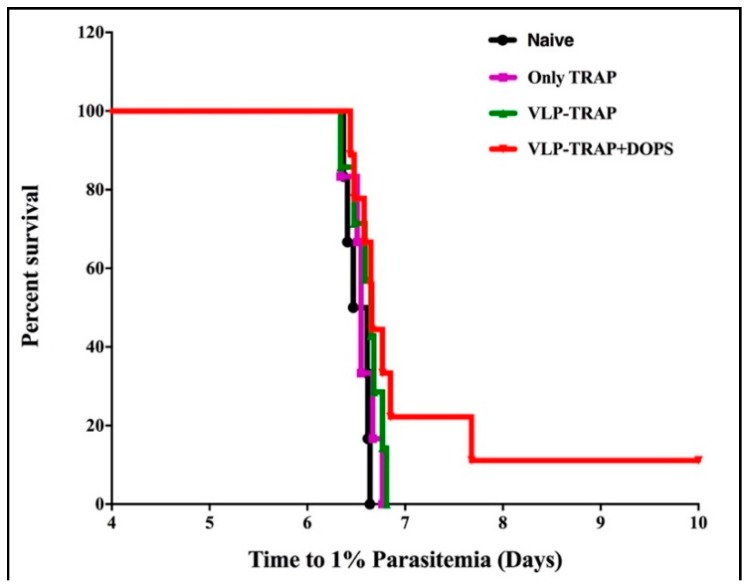
Kaplan–Meyer survival curve showing when the CD1 mice reached 1% or more of parasitemia or did not have any infected erythrocytes, which is considered to be sterile protection. The only group of mice showing some sterile protection was the group of mice vaccinated with PfTRAP-VLP plus DOPS (red line). Parasite load was counted daily in blood smears until 14 days after challenge and kept for another two weeks under observation to check for signals of disease. The group of mice immunized with PfTRAP-VLP plus DOPS conferred significant protection (*p* = 0.0001) when compared to the group control vaccinated with TRAP only.

To assess vaccine-induced protection, CD1 mice were infected with transgenic *P. berghei* expressing TRAP of *P. falciparum*, as described above. As seen in the Figure 7, mice vaccinated with CuMVtt-TRAP + DOPS showed a delay in parasitemia and even sterile protection was observed in some. To consider protection as sterile, mice should not have any erythrocytes infected until 14th day after challenge. The mice were observed for an additional two weeks to check for any signal of disease. At the end of each experiment the mice were sacrificed.

These data underscore the potency of CuMVtt-TRAP + DOPS as it is known that sterile protection is hard to achieve and usually only happens when using combinations of different antigens.

## 4. Discussion

In the present study, we assessed immunogenicity and protective capacity of PfTRAP formulated in DOPS or alum and studied the added value of coupling the antigen to CuMVtt-VLPs. To assess the protective capacity of the different vaccine formulations, we constructed a mutant *P. berghei* parasite by replacing its own TRAP by the TRAP of *P. falciparum*. This resulted in a malaria parasite able to replicate in mice but expressing TRAP of *P. falciparum.* Our results clearly show that PfTRAP conjugated to VLPs and formulated in DOPS induces the best T cell responses and the highest antibody responses dominated by protective isotypes. The same vaccine candidate also induced best protection in BALB/c inbred and CD1 outbred mice.

Most currently used adjuvants, such as Alum or MF59, are unable to promote strong Th1 responses but rather enhance Th2 responses. However, CTL responses usually go together with Th1 responses which therefore are not promoted by this type of classical adjuvants. Here we show that DOPS is able to promote both strong Th1 and CTL responses as well as the formation of IgG2a and IgG2b subclass antibodies. Phosphatidylserines are an indicator of cell death as they are expressed on the outer surface of apoptotic cells. As a consequence, they are categorized as prominent danger-associated molecular patterns (DAMPs). Whilst specific mechanisms of the adjuvant property of DOPS remain to be further elucidated, it will be interesting to further study this “danger signal” activity and elucidate its mechanism compared to alum. Indeed, it has been hypothesized in a recent report that a possible mechanism of alum relates to the induction of DAMP’s released by viable cells after phagocytosis of aluminum-based adjuvants [46].

VLPs, in particular VLPs loaded with RNA such as CuMVtt, are potent inducers of IgG responses dominated by the IgG2a isotype as well as Th1 and CTL responses. The high IgG responses are due to the repetitive nature of the VLP-surface: pathogen-associated molecular patterns (PAMPs), while the IgG2a and Th1/CTL responses are caused by the RNA spontaneously packaged by the VLPs during expression in *E. coli*. As such, a rational approach was sought in combining drug delivery concepts (i.e., VLP) in combination with second generation adjuvant (i.e., immunomodulators such as DOPS) functioned to provide a means by which immune activation could be better achieved for a vaccine to confer enhanced protection.

Strikingly, DOPS was not only able to significantly increase the IgG2a responses but also Th1 and CD8^+^ T cell responses. The latter may be of particular interest, as it is notoriously difficult to induce CD8^+^ T cell responses, in particular in humans. It will therefore be of interest whether these results can be confirmed in human clinical studies. This would not only impact the design of antiparasitic vaccines such as described here but extend to the field of cancer vaccination, where lack of efficient induction of tumor-specific CD8^+^ T cells remains a well-known problem.

## Supplementary Materials

The following are available online http://www.mdpi.com/2079-9721/6/4/107/s1, Figure S1: Strategy to generate a chimeric *P. berghei* parasite line expressing *P. falciparum* TRAP. Figure S2. Genotype analyses of the chimeric *P. berghei* parasite line expressing *P. falciparum* TRAP. Figure S3. Phenotype analyses and fitness of the chimeric *P. berghei* parasite line expressing *P. falciparum* TRAP. Figure S4. In vivo imaging. Table S1. Primers for genotyping the transgenic parasite line. Table S2. Primers for generation of DNA construct. Table S3. Oocyst and sporozoites production for PfTRAP(r)_PbTRAP_ transgenic parasite (line 2632 cl1) in comparison to the WT-GFP: Luc *P. berghei* (line 676m1 cl1).
