# Supplementary material for: DOPS Adjuvant Confers Enhanced Protection against Malaria for VLP-TRAP Based Vaccines
Source: Diseases. 2018 Nov 21;6(4):107. doi: 10.3390/diseases6040107 (PMC6313579; doi:10.3390/diseases6040107)
Supplement: Supplementary file 1 [file diseases-06-00107-s001.pdf]

Supplementary

DOPS adjuvant confers enhanced protection against malaria for VLP-TRAP based vaccines

Gustavo Cabral-Miranda[1,2*,a], Ahmed M. Salman[1,a], Mona O. Mohsen[1], Federico L. Storni[2], Elisa S. Roesti[2], Murray A. Skinner[3], Matthew D. Heath[3], Matthias F. Kramer[3], Shahid M. Khan[4], Chris J. Janse[4], Adrian V.S. Hill[1], Martin F Bachmann[1,2*]

[1] The Jenner Institute, Nuffield Department of Medicine, Centre for Cellular and Molecular Physiology (CCMP); University of Oxford; UK

[2] Immunology, RIA, Inselspital, University of Bern, Switzerland

[3] Allergy Therapeutics (UK) Ltd. Dominion Way, Worthing BN14 8SA, United Kingdom

[4] Department of Parasitology, Leiden University Medical Center, Albinusdreef 2, 2333 ZA Leiden, The Netherlands.

a Equal contribution as first author.

Correspondence may be addressed:

* Martin Bachmann - martin.bachmann@me.com

* Gustavo Cabral de Miranda - gcabral.miranda@gmail.com

Immunology, RIA, Inselspital, University of Bern, 3010; Switzerland; Phone +41 (0) 31 632 60 45 ; Fax: +41 (0)31 381 57 35

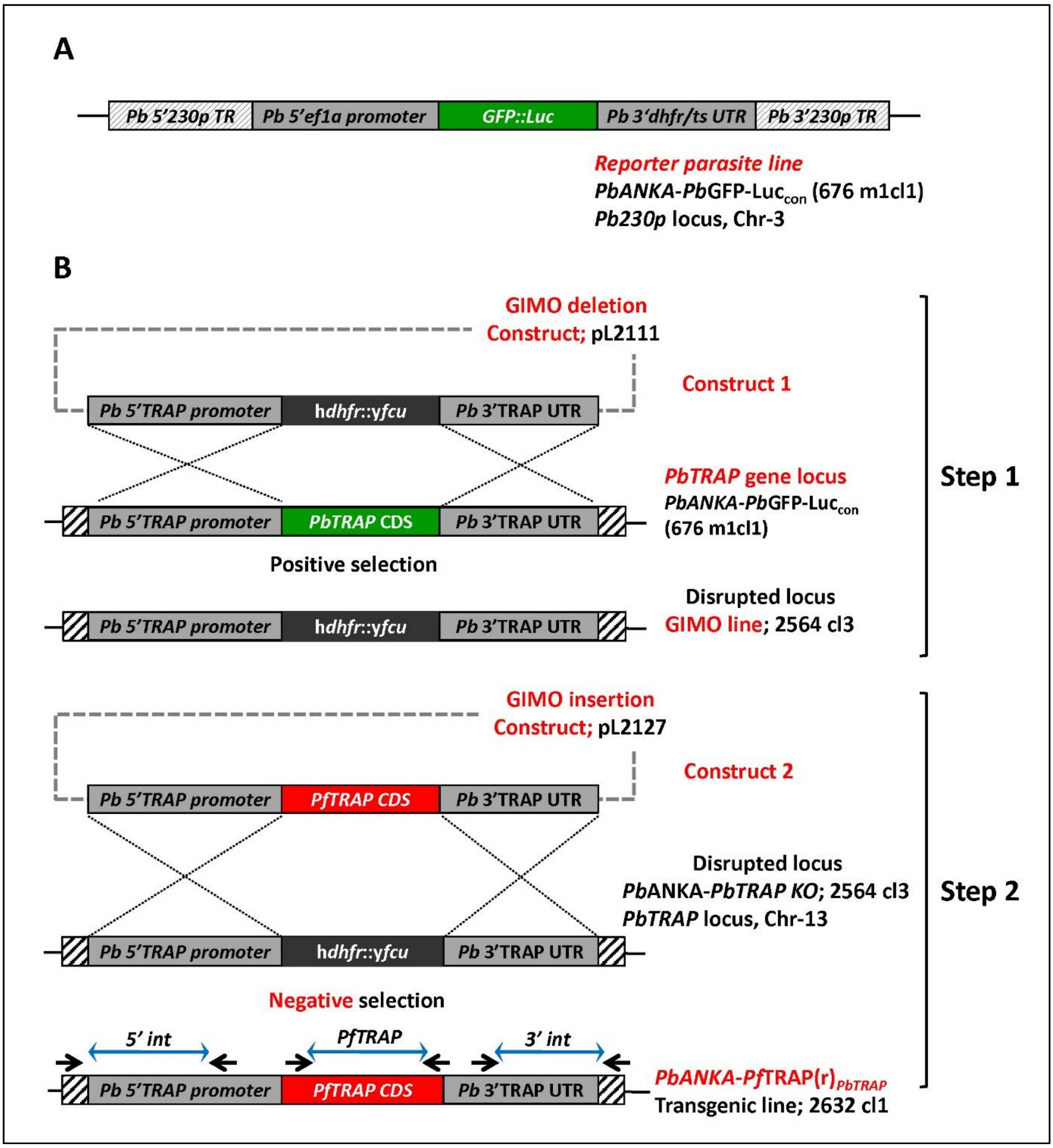

Figure S1. Strategy to generate a chimeric *P. berghei* parasite line expressing *P. falciparum* TRAP. A.Schematic representation of the transgenic *p230p* locus of the reporter *Pb*ANKA parasite line *PbGFP-Luc*$_{ceef1\alpha}$ (676m1cl1) which used to generate the single replacement gene [SRG] chimeric parasite line (see **B**). This reporter line expresses a fusion protein of GFP and firefly luciferase (LUC-IAV) under the constitutive *Pbeef1a* promoter and is selectable marker (SM) free. The reporter-cassette is integrated into the neutral *p230p* locus in chromosome 3. **B.** Schematic representation of the generation of the chimeric line PbANKA-PfTRAP(r)$_{PbTRAP}$ (line 2632 cl1) where the GIMO deletion-construct (construct 1; pL2111) is used to replace the *Pbtrap* coding sequence (CDS) with the positive/negative selectable maker (SM; h*dhfr::yfcu*) cassette, resulting in the generation of the *Pbtrap* GIMO line (*Pb*ANKA-*Pb*TRAP GIMO; line 2564 cl3) after positive selection with pyrimethamine. Construct 1 targets the *Pbtrap* gene by double cross-over homologous recombination. **S*tep* 2**: The GIMO insertion-construct (construct 2; pL2127) is used to replace the SM in the *Pbtrap* GIMO line with the *Pftrap* CDS after negative selection using 5-fluorocytosine (5-FC), resulting in the transgenic line PbANKA-PfTRAP(r)$_{PbTRAP}$ (line 2632 cl1). Construct 2 integrates by double cross-over homologous recombination using the same targeting regions (TRs) employed in construct 1, resulting in the introduction of the *Pftrap* CDS under the control of the *Pbtrap* gene

promoter and transcriptional terminator sequences and removal of the SM. Black arrows: location of primers used for diagnostic PCR (see Fig. S1-B).

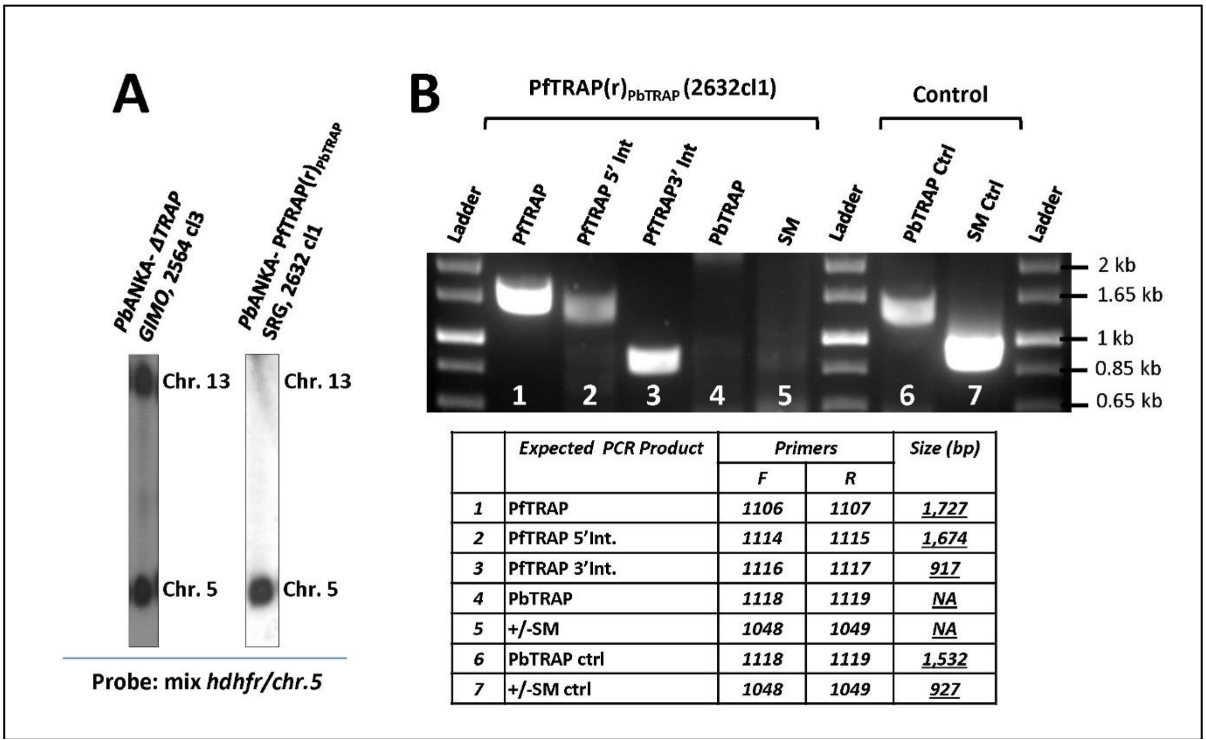

	Expected PCR Product	Primers F	Primers R	Size (bp)
1	PfTRAP	1106	1107	1,727
2	PfTRAP 5'Int.	1114	1115	1,674
3	PfTRAP 3'Int.	1116	1117	917
4	PbTRAP	1118	1119	NA
5	+/-SM	1048	1049	NA
6	PbTRAP ctrl	1118	1119	1,532
7	+/-SM ctrl	1048	1049	927

Figure S2. Genotype analyses of the chimeric *P. berghei* parasite line expressing *P. falciparum* TRAP. A.Genotype analysis of Single Replacement Gene [SRG] chimeric parasites (PbANKA-PfTRAP(r)$_{PbTRAP}$; line 2632 cl1) and their intermediate GIMO mother-line (2564 cl3) by Southern analysis of chromosomes (chr.) separated by pulsed-field gel electrophoresis (PFGE) and by diagnostic PCR analysis. **Left panel:** Hybridisation of chr. of line 2564 cl3 with a mixture of two probes: one recognizing h*dhfr* and a control probe recognizing chr-5 *c*onfirms integration of construct pL2111 (Fig. S2-A) into the *Pbtrap* gene on chr. 13. In addition, this probe hybridizes to chr. 5 (Fig. S2-A). **Right panel:** The correct integration of the *PfTRAP* expression construct (pL2127; Fig. S1) into the GIMO locus was confirmed by showing the removal of the h*dhfr::yfcu* selectable marker (SM) cassette in the cloned chimeric parasite line 2632 cl1. The southern blot is hybridized with a mixture of two probes: one recognizing h*dhfr* and a control probe recognizing chr-5. **B.** Genotype analysis by diagnostic PCR analysis of the chimeric parasite line 2632 cl1 (left panel) confirms correct integration of the *PfTRAP* expression cassette. Correct integration is shown by the absence of the h*dhfr*::*yfcu* SM and the *Pbtrap* CDS, the presence of the *PfTRAP* CDS, and the correct integration of the construct into the genome both at the 5′ and 3′regions (5′int and 3′int; see Figure S1 for primers locations). Primers sequences used are shown in Table S2, while the expected PCR product sizes and the primer numbers are listed in the table below the PCR analysis.

A

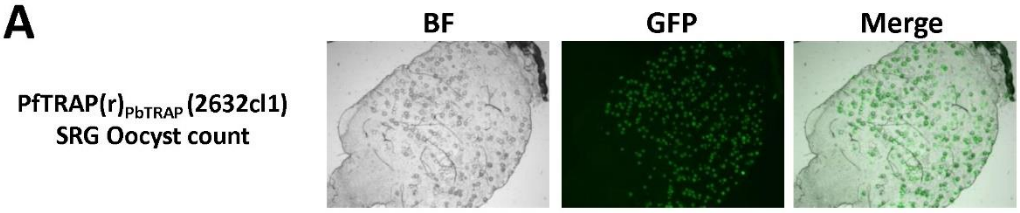

Line #	Oocyst no. per mosquito	Sprozoite no. per mosquito
	Mean±SD	Mean
2632 cl1	238±23	19,500

B

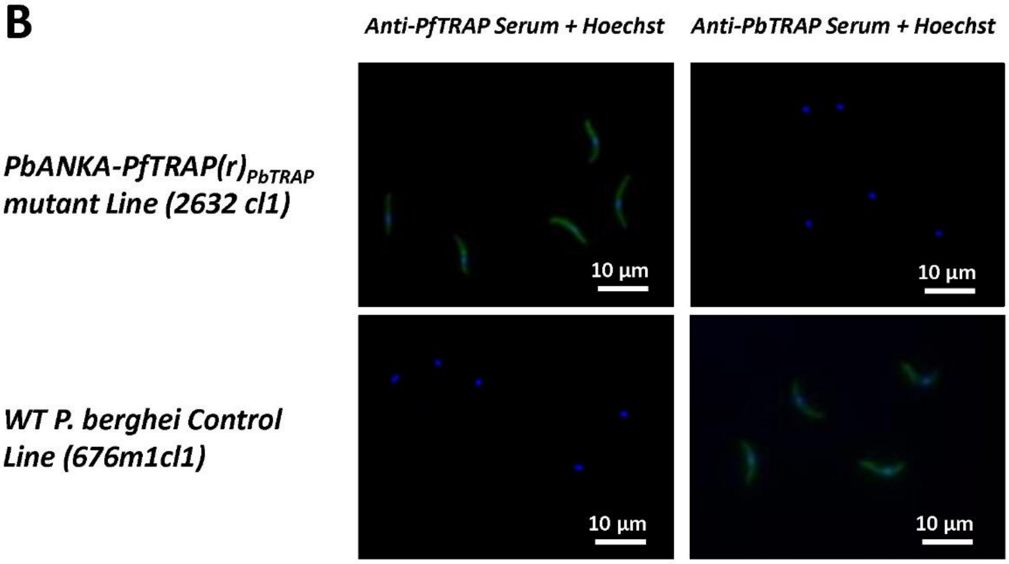

C

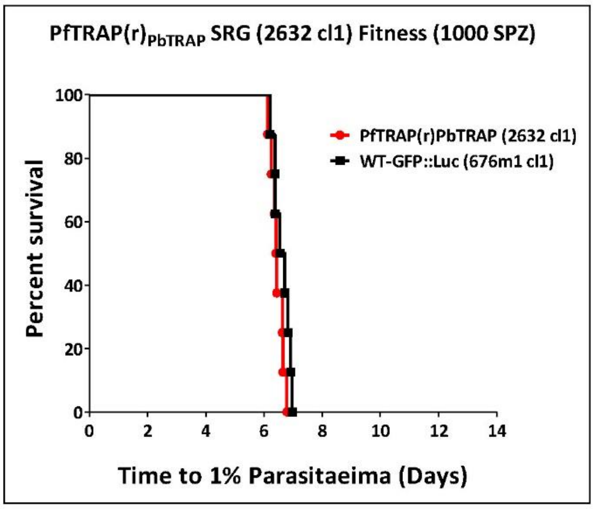

Figure S3. Phenotype analyses and fitness of the chimeric *P. berghei* parasite line expressing *P. falciparum* TRAP. A. Mosquito stage (midgut oocysts) expressing of GFP under the constitutively active *Pbeef1α* promoter. B. Immunofluorescence analysis demonstrating *PfTRAP* antigen expression in sporozoites of the chimeric line 2632 cl1. Salivary-gland sporozoites were stained with anti-*PfTRAP and anti-PbTRAP* antibodies containing sera from mice vaccinated against PfTRAP or PbTRAP (Alexa Fluor 488, green; nuclear staining with Hoechst-33342). As a control, wild-type (WT; line 676m1 cl1) *P. berghei* sporozoites were stained with the same antibodies containing sera. Merged images of the different channels are shown for both chimeric and WT *P. berghei* sporozoites. C.

Fitness assessment of sporozoites the chimeric line 2632cl1. Prepatent period in mice after injection of 10^3 sporozoites of line 2632 cl1 and of wild-type *P. berghei* parasites. The prepatent (i.e. the time to reach 1% parasitaemia) was similar in mice infected with 2632 cl1 and WT sporozoites (Log-rank (Mantel-Cox) Test; P-value 0.1254).

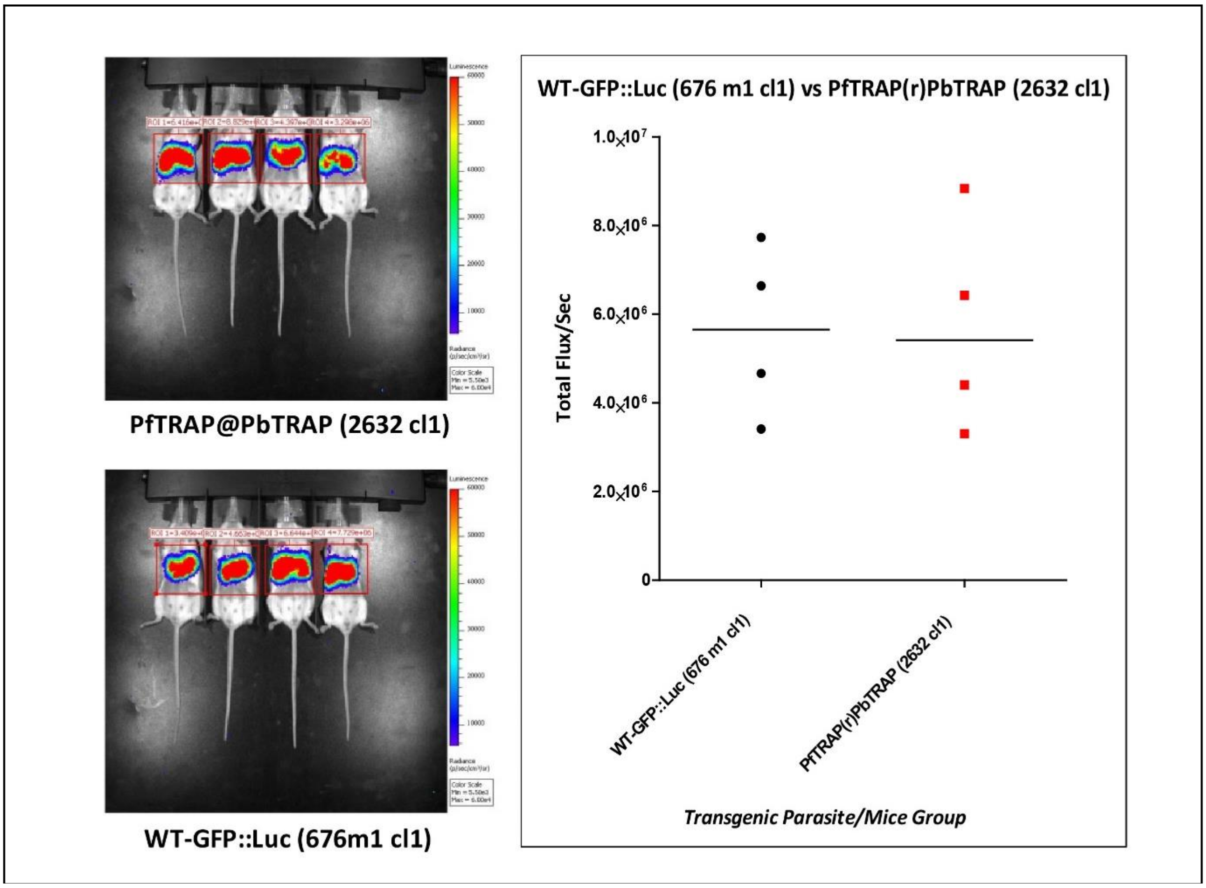

Figure S4. *In vivo* imaging. Liver loads in naïve mice that were challenged with transgenic sporozoites were quantified by measuring luminescence levels at 44 hours after infection using the IVIS 200 system. Results are presented as the total flux measured per second. Left panel: [A] WT-GFP::Luc (676ma cl1) control and [B] PfTRAP(r)PbTRAP, (2632 cl1). Right panel: Quantification of the bioluminescence signal emitted from infected mice in each group measured as total flux/sec (no significant difference between the two groups, P-value 0.9374).

Table S1. Primers for genotyping the transgenic parasite line.

Primer No.	Descriprtion	Primer sequences
1048	hDHFR-yFCU (+/-SM) F	ATCATGCAAGACTTTGAAAGTGAC
1049	hDHFR-yFCU (+/-SM) R	CATCGATTCACCAGCTCTGAC
1106	PfTRAP Int. F	ATGAATCATCTTGGGAATGTTAAATAT TTAGTC
1107	PfTRAP Int. R	ATTTAATTCCACTCGTTTTCTTCAGG
1114	PfTRAP@PbTRAP 5'Int.	AAATTGCCCCCTTTTTTGTGTTC
1115	PfTRAP@PbTRAP 5'Int.	TTGCACATCTCTACCATTAACTAG
1116	PfTRAP@PbTRAP 3'Int. F	AACACCCTATGCCGGAGAAC
1117	PfTRAP@PbTRAP 3'Int. R	AATGACTCCAGACATAATAACACAGA TATG
1118	PbTRAP F	ATGGCTCAGGAAGTATTGGTC
1119	PbTRAP R	ACCTATGCATCCAATTATAGCTAATC

Table S2. Primers for generation of DNA construct.

DNA Construct	Primer No.	Primer sequences *	Restriction sites	Fragment size (bp)	Descriprtion
pL2111	1145	catgggcccGGAAATTGTCTTACC CATATTATTCCTAC	ApaI	1494	PbTRAP 5′-UTR promoter sequence, F
	1146	atctgcaggttaacGAAAGGGAAAA TGGGCAAATTATGTGTC	PstI + HpaI		PbTRAP 5′-UTR promoter sequence, R
	1147	ccggggtaccggatccCATATATATC TAGATGATTATTCTTATGTTA C	KpnI + BamHI	637	PbTRAP 3′-UTR sequence, F
	1148	ataagaatgcggccgcAACTTAAGAG TATTATTTTTGTTTCG	NotI		PbTRAP 3′-UTR sequence, R
pL2127	1156	tatcctgcaggATGAATCATCTTGG GAATGTTAAATATTTAGTC	SbfI + PstI	1747	PfTRAP, F
	1157	gtaggatccATTTAATTCCACTCG TTTTCTTCAGG	BamHI		PfTRAP, R

* Red color: Restriction site sequence.

Table S3. Oocyst and sporozoites production for PfTRAP(r)$_{PbTRAP}$ transgenic parasite (line 2632 cl1) in comparison to the WT-GFP::Luc *P. berghei* (line 676m1 cl1).

Parasite descirption	Line number	Oocyst no. (Mean ± SD)	Sporozoites no. (Mean ± SD)
WT P. berghei	676m1 cl1	116±22	22,800
PfCSP(r)$_{Pbcsp}$	2632 cl1	238±23	19,500
